# Gaze-based attention network analysis in a virtual reality classroom

**DOI:** 10.1016/j.mex.2024.102662

**Published:** 2024-03-15

**Authors:** Philipp Stark, Lisa Hasenbein, Enkelejda Kasneci, Richard Göllner

**Affiliations:** a University of Tübingen, Hector Research Institute, Europastraße 6, 72072 Tübingen, Germany; b Technical University of Munich, Chair for Human-Centered Technologies for Learning, Arcisstraße 21, 80333 München, Germany; c University of Regensburg, Institute of Educational Science, Universitätsstraße 31, 93053 Regensburg, Germany

**Keywords:** Network analysis, Eye tracking, Virtual reality, Gaze-ray casting, Visual attention, Graph theory, Gaze-based Attention Network Analysis

## Abstract

This article provides a step-by-step guideline for measuring and analyzing visual attention in 3D virtual reality (VR) environments based on eye-tracking data. We propose a solution to the challenges of obtaining relevant eye-tracking information in a dynamic 3D virtual environment and calculating interpretable indicators of learning and social behavior. With a method called "gaze-ray casting," we simulated 3D-gaze movements to obtain information about the gazed objects. This information was used to create graphical models of visual attention, establishing attention networks. These networks represented participants' gaze transitions between different entities in the VR environment over time. Measures of centrality, distribution, and interconnectedness of the networks were calculated to describe the network structure. The measures, derived from graph theory, allowed for statistical inference testing and the interpretation of participants' visual attention in 3D VR environments. Our method provides useful insights when analyzing students’ learning in a VR classroom, as reported in a corresponding evaluation article with *N* = 274 participants.

• Guidelines on implementing gaze-ray casting in VR using the Unreal Engine and the HTC VIVE Pro Eye.
• Creating gaze-based attention networks and analyzing their network structure.
• Implementation tutorials and the Open Source software code are provided via OSF: https://osf.io/pxjrc/?view_only=1b6da45eb93e4f9eb7a138697b941198.

## Graphical abstract

**Figure:**
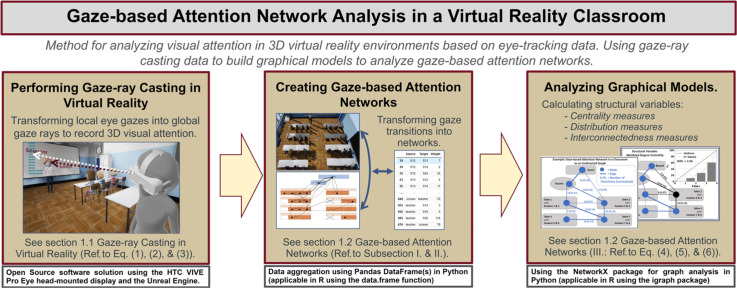

Specifications table

**Table:** 

Subject area:	Computer Science
More specific subject area:	Human-Computer Interaction, Virtual Reality, Eye Tracking
Name of your method:	Gaze-based Attention Network Analysis
Name and reference of the original method:	Ray Casting, Network Analysis
Resource availability:	**Hardware:** (1) HTC VIVE Pro Eye https://www.vive.com/us/product/vive-pro-eye/overview/ ***Software:*** (1) Unreal Engine https://www.unrealengine.com/de (2) SRanipal Unreal SDK https://developer.vive.com/resources/vive-sense/eye-and-facial-tracking-sdk/documentation/ (3) Python 3.11 + any IDE (4) Python package requirements (see requirements.txt): ▪ numpy https://numpy.org/ ▪ pandas https://pandas.pydata.org/ ▪ networkx https://networkx.org/ (5) Eye-tracking C++ scripts (see OSF) (6) Analysis pipeline in Python (see OSF) https://osf.io/pxjrc/?view_only=1b6da45eb93e4f9eb7a138697b941198*For an illustration of the VR environment and original experiment, see*http://vre-tuebingen.de.

## Method details

### Background and motivation for applying the method

Due to recent technological innovations, virtual reality (VR) has celebrated a rebirth in the consumer market, with immersive, head-mounted VR devices at affordable prices applicable in different fields of all our lives [1]. Specifically, recent developments in hardware, software, and design have resulted in VR applications being more frequently used in education and education research [2]. With VR, learning environments like virtual classrooms can be studied systematically, for example, to investigate classroom complexity [3], seating arrangements [4], or performance-related classroom behavior [5]. These developments embrace the possibility of uncovering aspects of learning that were previously difficult to study in these settings, such as visual attention. However, collecting and analyzing visual attention information in VR poses some challenges. A significant challenge involves acquiring relevant, high-quality eye-tracking data in dynamic 3D virtual environments. Aggregating this data to obtain meaningful indicators of learning and social behavior presents another complex challenge.

This method article presents a way to overcome these challenges and provides systematic step-by-step guidelines for measuring and analyzing human visual attention based on VR eye-tracking data. Our article is published alongside a corresponding empirical evaluation by Hasenbein et al. [5], where we evaluated our method with *N* = 274 students to investigate learning with simulated virtual classmates in a VR classroom. We examined gaze-based visual attention to investigate students’ performance, interest, and self-concept during a 15-minute teaching unit in a virtual classroom. The visual attention of these participants was analyzed based on eye movement data. More specifically, eye movements were obtained and analyzed using a VR device with a head-mounted display (HMD) and an integrated eye tracker [6]. For the experiment, the HTC VIVE PRO EYE head-mounted display (HMD), which operates with the Tobii eye tracker [7], was used. The Unreal Engine allows game developers to provide an immersive, interactive, and animated visualization of virtual learning environments for application in education research [2].

Given this VR experiment, the first challenge occurred when collecting eye-tracking information. In our study, we specifically focus on overt visual attention, the process of attentional shift using eye movements [8], which can be measured by simulated gaze movements in space. More specifically, overt visual attention towards an object is the intersection of a human's gaze ray with an object for a specific time. The integrated HMD eye tracker only reports participants’ local gaze directions, which does not consider the position and viewing direction. To analyze the focus points of visual attention and collect semantic information about the gazed objects, HMD, and eye tracker data must be combined and processed. This can be achieved by applying gaze-ray casting, where humans’ gaze direction is transported in the environment to observe where it hits. This method already exists for other game engines and eye-trackers. A more detailed review of this challenge can be found in Ugwitz et al. [9], [10], [11]. We faced the challenge that a simple software approach was missing for the Tobii Eye Tracker in combination with the Unreal Engine. Therefore, in the first part of our article, a software solution is provided that can be easily integrated into existing projects.

The second challenge occurred when deciding on the appropriate level of data aggregation. Experimental psychology offers a wide range of methods for processing eye-tracking data when investigating learning. This is usually based on determining the smallest eye movements, such as saccades and fixations, which provide insight into underlying cognitive processes [12]. However, this method does not include information about the environment or participants' guidance of visual attention. To incorporate semantic scene information into the analysis [13], methods like scan path analysis can be applied [8,14,15]. However, scan path analysis relies on specific distance measures or machine learning algorithms to compare their structures [8]. Another promising way to analyze this information can be achieved by creating graphical models [16], [17], [18], which we call gaze-based attention networks. The idea of using network representations is probably most prominent in social network analysis [19,20]. Our gaze-based networks represent participants’ gaze transitions between different virtual entities in the environment over a period of time. The structure of these networks follows the mathematical principles of graph theory [21,22], with objects in the environment treated as network nodes and gaze transitions treated as edges between them. This method has been applied for stationary eye tracking on a screen in previous research [23] concerning experimental and clinical psychology [20], [21], [22], mathematical problem-solving [23], or joint attention [24]. It allows to describe the composition and interconnectedness of the gaze-based networks using measures from graph and network theory. These measures, which we refer to as structural variables [25,26], allow us to describe the network structure of participants’ visual attention in 3D VR environments and statistically analyze and compare participants' visual attention.

To provide a rationale for the method, the following potential advantages of the approach can be highlighted. An Open Source software solution was coded for the Unreal Engine to quickly integrate it into existing projects by following the guidelines. Also, details are presented on how the data collection pipeline with gaze-ray casting can be extended and adjusted to the needs of specific projects. Our method of transforming gaze-based information into a transition network eliminates the need to compute eye movement events such as fixation and saccades, which can be challenging in 3D environments [27,28]. Besides minor data exclusion steps, the collected eye-tracking information can be processed without extensive data cleaning. This provides a quick way to aggregate gaze networks directly from the data collected by the gaze-ray casting pipeline.

Further, modeling eye-tracking data as gaze-based attention networks could be more intuitive to interpret for applied researchers since they can be easily visualized. Data aggregation comes with information loss and determines the possibilities of analyzing and interpreting the data, so the level of data aggregation must be chosen appropriately for the research interest. The network structures contain semantic information, including participants’ reactions to virtual social actors. Especially on this level of visual attention aggregation, gaze transitions between social actors can provide meaningful information. Since empirical studies in the social sciences are interested in interpretable measures, structural variables offer a valuable and comprehensible way for statistical testing.

### Structure of the article

This article is structured in four parts: First, guidelines and instructions are provided on implementing gaze-ray casting using the Unreal Engine to record gaze target information from users during a virtual reality (VR) experience (see Section 1.1). Second, we show how to transform the obtained gaze target information into a gaze-based attention network, compute structural variables of the networks, and interpret them in the case of visual attention (see Section 1.2). Third, the performance of the data pipeline in Python was evaluated, and samples of the code in the programming language R were provided. We hope that this can increase the applicability and reproducibility of the method, especially for researchers not familiar with Python (see Section 1.3). Last, some general considerations for implementation and application were provided (see Section 1.4). Additional lessons learned during the implementation are described in the *tips for application.*

Further instructions, illustrations, and implementation details are given at OSF: https://osf.io/pxjrc/?view_only=1b6da45eb93e4f9eb7a138697b941198*.* The OSF repository structure corresponds to the article structure, with additional information within the Readme.md in each section folder. To facilitate the reproducibility of the method, the code locations are referred to throughout the text by referencing the OSF project (Ref. to OSF). Special technical terms used in the method are explained in Table 1.

**Table 1:** Overview and explanation of technical terms used in this article.

Time point	Game engines work with a specific framerate in which they update the environment. A time point is one tick or update frame in the virtual environment, considering that time intervals between two points do not differ significantly. The tick rate is based on device performance (on average, every 20 ms).
Local gaze direction	A normalized vector of the HMD eye-tracker is expressed in the coordinates of the local coordinate system of the VR headset.
Global gaze direction	A vector that starts at the cyclopean eye and points into the virtual environment. This vector is stated in unreal units (uu), equal to 1 centimeter in real-life distance.
Gaze target	The virtual object is hit by the lengthened global gaze direction where the gaze position is currently located (stated in uu).
Object of interest (OOI)	Closely related to the term Area of Interest (AOI), which describes a segment of a stimulus space. OOIs are the objects of a preselected set of potential gaze targets considered in the analysis.
Gaze-ray casting	A technique to obtain gaze target (information) using the global gaze direction and object location provided by the Game Engine. See Section 1.1. Gaze-ray Casting in Virtual Reality for detailed information.
Gaze transition	A gaze shift between two successive OOIs. More precisely, the gaze movement between the last detected gaze location on one object and the first detected gaze location on the next.
Player / User	When describing functions and algorithms in the Unreal Engine, the player is used to describe the virtual character created in the 3D space as a projection of the user's position in the room. User refers to the person who is using the VR device.

### Gaze-ray casting in virtual reality

Ray casting is known and used primarily as an interactive technique in VR environments for target selection with a controller [29]. Gaze-ray casting is based on a similar idea: the direction of a human's gaze is considered a ray. Starting at the position of the cyclopean eye, the middle point between both eyes, the gaze is projected into the virtual environment, where it hits a specific location or, in other words, a gaze target [30,31]. The gaze-ray casting technique detects the gaze target directly during the VR experience. It enabled us to collect various information, like the label or position of the gaze target or the distance between the player and the target [32].

This has an advantage compared to remote or real-world mobile eye trackers. In remote eye trackers, gaze targets must be annotated separately by labeling the pictures or videos on the screen. This is even more complicated in real-world mobile eye trackers because of the user's free movement in a 3D space see [33]. In contrast, when using an immersive VR [34], a 3D environment experienced through a head-mounted display, the game engine renders all of the virtual scenery. This means complete information about objects’ location and shape is always available. Therefore, the gaze target is just an intersection of humans’ gaze rays with the polygon surface of the closest object in the virtual space, referred to as the gaze intersection point [35]. The gaze-ray casting technique is also independent of detecting and calculating eye movement events, like fixations and saccades [27]. The only information received is about which object a user is looking at a time point, and no eye movements need to be calculated.

The implementation of gaze-ray casting in VR with the Unreal Engine (UE) can be divided into five steps, which should be performed sequentially. In addition to an implementation tutorial (Ref. to OSF: 1-1_Gaze-rayCastinginVirtualReality/Readme.md), a detailed description of each step is given below.

I. Enabling eye tracking in the Unreal Engine using the SRanipal SDK.
II. Creating an “eye-tracking” Actor to collect the local gaze vectors.
III. Transforming local gaze directions into global gaze directions.
IV. Projecting the global gaze vector into the environment using a ray casting function.
V. Collecting gaze target information in the eye-tracking Actor and saving it in a data file.

#### Enabling eye tracking in the Unreal Engine using the SRanipal SDK

To collect eye and gaze data with the HTC Vive Pro Eye in the UE, the provided VIVE software was used [36]. As described in the website's documentation, the SDK was integrated into an Unreal project and enabled access to all eye-tracking variables recorded by the integrated eye tracker.

#### Creating an “eye-tracking” Actor to collect the local gaze vectors

A combination of C++ scripts and Unreal Blueprints was used to create a data collection pipeline for the project. Unreal blueprints are node-based interfaces to create gameplay elements in UE that grant easy access to already implemented functions. To further process the gaze data, a new C++ Actor Class (Ref. to OSF: 1–1_Gaze-rayCastinginVirtualReality/EyeTracker.h/.cpp) and a corresponding Actor Component Blueprint (“BP_EyeTracker”) were created, where all necessary calculations were implemented. In the C++ files, the local gaze directions were stored and transformed into UE vector objects to be further processed as gaze vectors in our EyeTracker Blueprint class (Ref. to OSF: 1-1_Gaze-rayCastinginVirtualReality/Readme.md).

*Tips for application:* Whenever an Unreal-type vector is created in a C++ script, the variable can be accessed in the blueprint of the connected Actor. The eye-tracking data collection from the eye tracker could also be integrated into already existing Actors in the virtual environment, but the separate data collection Actor was a convenient way to add eye tracking into already existing projects.

#### Transforming local gaze directions into global gaze directions

Continuing in the blueprint component, the location and orientation of the EyeTracker Actor had to be aligned with the player's head location and orientation. Therefore, the EyeTracker Actor was aligned with the Pawn, the main Actor in UE.

As a next step, the local gaze vector, received from the C++ script, was transformed into a global gaze direction. The forward head direction of the player could be accessed by recording the player's perpendicular head vector. This normal vector is pointing forward perpendicular to a plane describing the front or face of the player. When a user moves their head, this vector moves in sync. Simultaneously, this forward vector represented the head direction of the HMD headset but also the x-axis of the local (eye tracker) coordinate system.

##### Tips for application

In the blueprint, the function <<Get Forward Vector>> was used to get the forward vector with the player rotation as input.

**Fig. 1:**
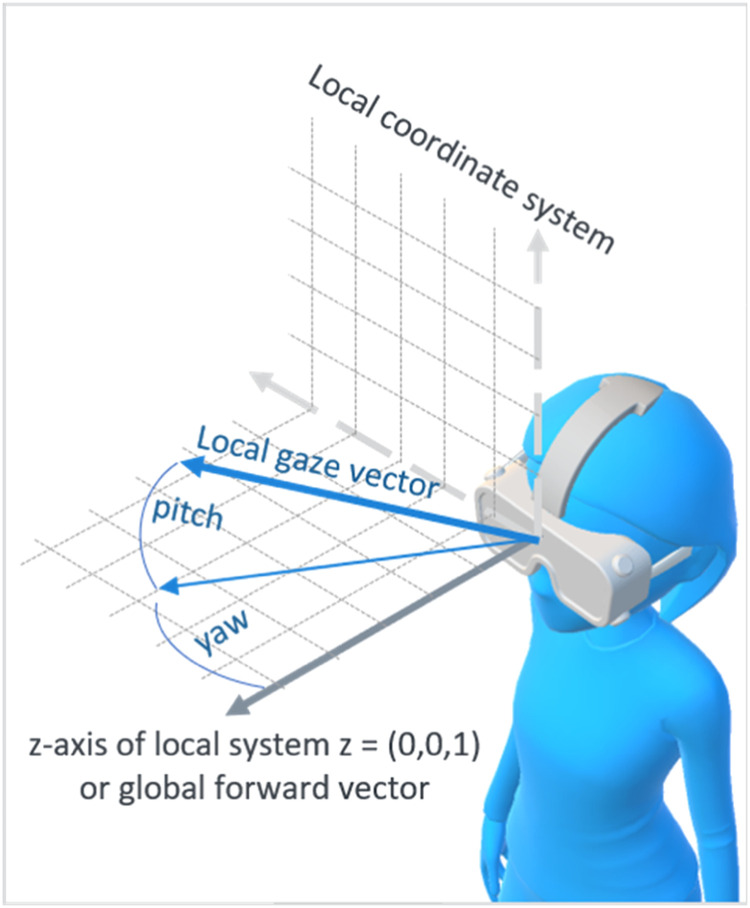
How to calculate pitch and yaw using the local gaze vector from the local coordinate system of the Tobii Eye Tracker.

The forward vector had to be rotated to align locally with the local gaze vector. To perform the rotation, yaw (i.e., the head rotation angle in degree to the left or right from a vertical axis) and pitch (i.e., the angle in degree at which one is looking up or down) were calculated. This method could be used because angle-based rotations are independent of the coordinate system and its units (see Fig. 1).

Euclidian geometry was used to calculate the yaw and pitch angle. As a reference vector, the forward vector $f=\left(x_{f},y_{f},z_{f}\right)=\left(0,0,1\right)$ was used given in unreal units (uu), where 1uu is equal to 1 centimeter. With the normalized gaze vector $g=(x_{g},y_{g},z_{g})$ and the flat 2D gaze vector $g_{flat}=\left(x_{g},0,z_{g}\right)$, the yaw angle in degree was calculated by

(1) $$yaw=-\;\cos^{-1}\left(\frac{z_{g}}{\sqrt{x_{g}^{2}+z_{g}^{2}}}\right)*\frac{180}{\pi *sgn\left(x_{z}\right)},\;with\;yaw\in [-180^{\circ },\;180^{\circ }].$$

The minus one and the signum function in Eq. (1) introduced a change in the orientation of the coordinate system. The pitch angle was calculated by

(2) $$pitch=\cos^{-1}\left(\frac{x_{g}^{2}+z_{g}^{2}}{\sqrt{x_{g}^{2}+z_{g}^{2}}}\right)*\frac{180}{\pi *sgn\left(y_{z}\right)},\;with\;pitch\in [-180^{\circ },\;180^{\circ }]\;.$$

With both angles from the Eqs. (1) and (2), a vector rotation (<<RotateVector>>) was performed on the forward vector f to create $f_{rotated}$. Lastly, the global gaze vector was computed starting at the players’ head location $h_{location}$ in uu as

(3) $$g_{global}=h_{location}+\left(f_{rotated}*k\right),\;with\;g_{global}\in R^{3}\;and\;k\;\in R.$$

In Eq. (3), k represents the length of the gaze vector in uu. The global gaze vector $g_{global}$ is then also stated in unreal units. After calculating the global gaze direction, this vector was used to perform gaze-ray casting.

##### Tips for application

Independently of the environment, the value of k in Eq. (3) can be set very large (we set k=25000uu for our experiments) because the ray cast will stop when it hits the first object. One problem when working with the Tobii eye tracker and the UE was that these two software presented gaze and head direction on different spatial coordinate systems. The Unreal Engine has a left-handed coordinate system, with positive x pointing forward (clockwise roll rotation), y pointing right (clockwise pitch rotation), and z pointing upwards (counterclockwise yaw rotation) [37]. In contrast, gaze information of the Tobii eye tracker was given in a right-handed coordinate system with z pointing forward, x pointing to the left, and y pointing up. The presented formulas for calculating yaw and pitch (Ref. to Eqs. (2) and (3)) already include the coordinate change, so pitch and yaw could be used directly for vector rotation.

#### Projecting the global gaze vector into the environment using a ray-casting function

To get information about gaze location and gaze target, functions from the Kismet System Library were used. To perform the gaze-ray casting, either <<LineTraceByChannel>> or <<LineTracForObjects>> can be used. The two functions differ only in considering different object types as hit objects. Both blueprint functions perform ray casting automatically by taking a starting position, namely the player's head location, and an end position, namely the global gaze vector ($g_{global}$). Useful output variables of these functions are the name of the gaze target (*Hit Component*, i.e., a virtual object as a string), the 3D location of the gaze hit (*Location or Impact Point in uu),* and the distance from the player to the hit object (*Distance in uu*).

*Tips for application:* One important aspect is that any line trace function only returns the first hit object. Therefore, one needs to ensure that no other (potentially invisible or hidden) objects are in the player's line of sight. To this end, all colliders of hidden objects had to be disabled, while at the same time, collision for all objects one wanted to track had to be enabled such that the line trace could hit our Objects of Interest (OOIs).

#### Collecting gaze target information in the eye-tracking Actor and saving it in a data file

The gaze-ray casting output variables were stored in the blueprint for each time point and accessed via the C++ script. Together with other eye-tracking information from the integrated eye tracker, the gaze-ray casting variables (gaze location, gaze target, ray distance) were saved into a data frame marked with the timestamps. The resulting dataset was stored as a CSV file at a predefined project location.

##### Tips for application

To follow our code structure: In the C++ file (Ref. to OSF: 1-1_Gaze-rayCastinginVirtualReality/EyeTracker.cpp), the gaze vector was created at line 71. Then, the gaze-ray casting was performed in the blueprint, and its output was stored starting from line 103.

### Gaze-based attention networks

The previous pipeline collected gaze target information from users during the VR experiences. The obtained information (gaze target and time stamp) could then be used to analyze data via very different means. In our virtual classroom study [5], the gaze target information was transformed into networks, providing a flexible approach to analyze and visualize gaze-based visual attention [18,[38], [39], [40]]. The gaze-based networks contained aggregated information about participants’ visual attention in the virtual environment, represented in a network structure. Thus, we call them gaze-based attention networks. Concepts from mathematical graph theory and network analysis were used to calculate descriptive variables that reveal information about the network structure [21], which we call structural variables. The networks represented by the structural variables were then associated with social comparison and learning by performing statistical inference testing [41].

**Fig. 2:**
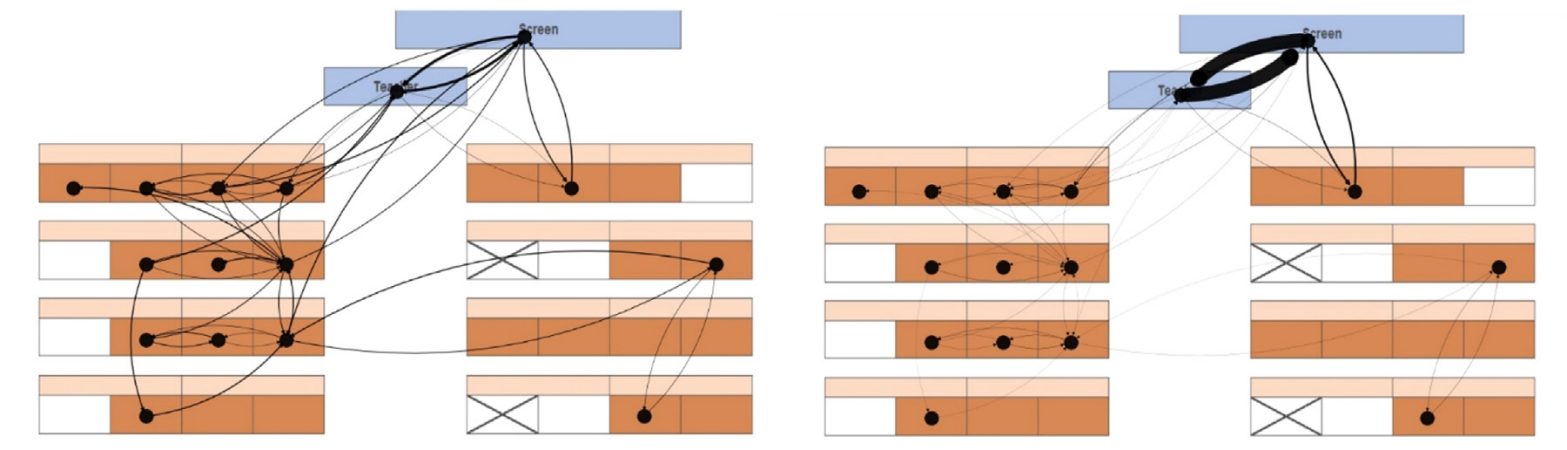
Visual representation of gaze-based attention networks from two participants in a top-down view on the virtual classroom. All OOIs are the teacher and board in blue and the positions of the virtual peer learners at their table in orange. Frequencies of gaze transitions between gazed OOIs are illustrated by the line width of the edges.

In the virtual classroom study, participants spent 15 min listening to a lecture about computational thinking. Gaze targets were objects in the environment from which the gaze-ray casting information was collected. One visual attention network was built per participant. Each network structure consisted of nodes, which were the virtual peer learners, the teacher, and the board in the classroom (our OOIs). The nodes were connected by edges, representing the frequencies of participants’ gaze transitions between the OOIs. The more often a participant switched visual attention from one OOI to another, the larger the edge weight between two OOIs, indicating a stronger connection. As a result, each network represented a bidirectional, weighted graph of overt visual attention distribution in a virtual classroom. Example networks for two participants are shown in Fig. 2. Performing network analysis with VR gaze data and computing structural variables required three (pre-) processing steps described in the following sections:

I. Aggregating raw gaze-target information into gaze transition datasets.
II. Creating gaze-based attention networks from gaze transition datasets.
III. Computing structural variables to describe gaze-based attention networks.

#### Aggregating raw gaze-target information into gaze transition datasets

As a first step, the data had to be cleaned. This can be done using any reliable procedure and considering other eye-tracking variables like pupil size to identify missing data and artifacts e.g. [33,42,43] (Ref. to OSF: 1-2_GazeBasedAttentionNetworks/ fl1_preprocessing.py). To conduct the network analysis, as explained in the subsequent steps, only the time and gaze target variables were needed.

##### Tips for application

The gaze-ray casting pipeline always reports a gaze target as long as a valid head direction is recorded. It is important to exclude missing values coded as placeholders for missing values (like −1). The calculation of pitch and yaw is also performed with incorrect values. If outliers and missing data are not excluded in a separate step, the global gaze direction might be incorrect, and the gaze target might be false.

**Fig. 3:**
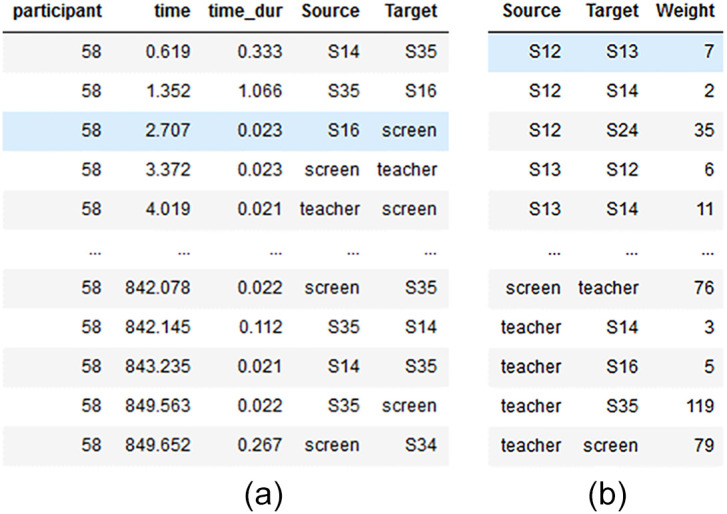
(a) Transition data frame, with transitions between starting and landing OOI (form Source to Target), marked with the starting time of the transition and the transition duration. The participant variable indicates that such a data frame is created separately for each participant. (b) An adjacency-like pandas edge list data frame. Serves as input for the networkx function which creates the graph object.

After cleaning the raw data, a new data frame was created for each participant, which consisted of all gaze transitions between OOIs during one experimental session. Changes in OOIs were stored in the new transition dataset by iterating through each row of the raw dataset. Thus, if the gaze target was not the same as in the previous line, the following information was stored in the transition dataset: the time stamp at the beginning of the transition, the transition duration, the transition starting object, and the transition landing object. An example of a transition data frame is shown in Fig. 3a.

As a last step, some transitions needed to be excluded. We decided on an upper threshold for the maximum transition duration. The time duration variable was used to filter the dataset for longer durations (Ref. to OSF: 1-2_GazeBasedAttentionNetworks/fl2_transition.py).

##### Tips for application

Excluding longer transitions seems necessary to ensure that only direct transitions are counted. If a transition duration was too long, it was likely that no direct shift was observed but rather many shifts between objects that were not considered OOIs.

#### Creating gaze-based attention networks from gaze transition datasets

Now, the datasets were used to aggregate the input variables necessary to create graph objects with the networkx [44] package. This Python package offers useful default functions, which can be customized for later analysis. With <<networkx.from_pandas_edgelist()>>, a graph object was created directly from an adjacency-like data frame. This required creating a new dataset containing a source and target variable. In this new dataset, the source and target variables stored information about the connected nodes, while a third (weight) variable contained information about the strength of the edge connection. The weights were calculated to describe the total number of gaze transitions between respective OOIs. The weight variable held all edge information from a weighted graph by counting the total number of gaze transitions across all OOIs. An example of an input data frame is shown in Fig. 3b (Ref. to OSF: 1-2_GazeBasedAttentionNetworks/fl2_transition.py). As a result, one graph represented one gaze-based attention network for one participant during one experimental session. The networkx graph objects (<graph_name>.p) were visualized with function family around <<networkx.draw()>> and used for further analysis. (Ref. to OSF: 1-2_GazeBasedAttentionNetworks/main.py).

##### Tips for application

The gaze transition dataset was grouped by the source and the target variable and counted how often each combination occurred. Proceeding like this for the whole dataset, the number of transitions from each object to all others could be counted, using only one line of code. The complexity of the resulting graphs depended on the number of total OOIs (nodes) and the frequency of gaze transitions (edge weights). The size of the files was reduced significantly by saving networkx graph objects instead of CSV datasets. All transition datasets for our participants had an average size of 40 KB, while the stored graph files only had an average size of 1.3 KB.

#### Computing structural variables to describe gaze-based attention networks

To compare the network structures between participants, various measures can be computed to compare values between graphs e.g. [45,46]. Our selection of structural variables allowed networks to be compared statistically. All structural variables below can be assigned to one of these categories:

• *Centrality measures*
• *Distribution measures*
• *Interconnectedness measures.*

Centrality measures describe a node's importance or prominence within a network by assessing the number or weight of connections it holds with other nodes [47]. These calculations can be used to determine the importance of certain OOIs in relation to visual attention in the virtual environment. Distribution measures act as a proxy for understanding how visual attention is distributed among specific nodes compared to all others [26]. They provide a means to analyze the distribution of visual attention within a network. Interconnectedness measures focus on the connections between nodes in a network [48]. In gaze-based attention networks, they help to understand how OOIs are linked, i.e., which OOIs build subgroups with frequent gaze transitions. The example code on how to calculate the structural variables is provided in the repository (Ref. to OSF: 1-2_GazeBasedAttentionNetworks/fl3_calculate_graph_features.py).

### Centrality measure

**Fig. 4:**
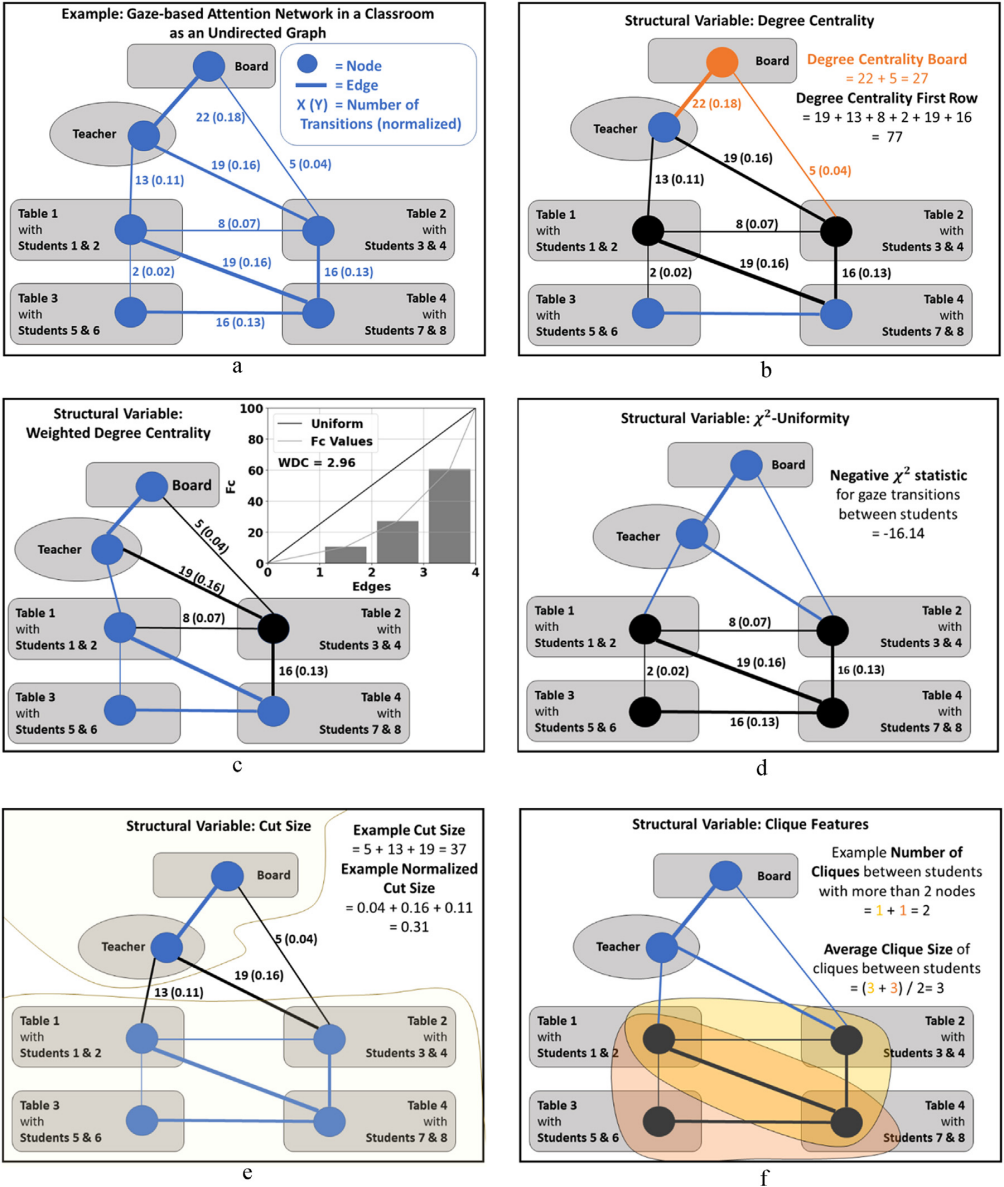
Examples of computing structural variables from an undirected graph. A scenario of gaze transitions in a classroom is shown with reduced complexity (fewer nodes) to create a gaze-based attention network for a participant. The example network has the same nodes and edges in all structural variable calculations. A larger display of the example images can be found in the Supplementary Material.

*Degree centrality* is a measure calculated as the sum of the weights of a node's incoming and outgoing edges. Previous studies have used this centrality measure to investigate visual attention [49]. It indicates the frequency with which a participant transitions toward a specific object. It is also possible to sum up the degree centrality for a group of OOIs. For comparing its value between participants (i.e., between graphs), one must ensure that one always considers the same group of nodes. To calculate degree centrality, the <<degree>> function of the network package was used. Moreover, its functionality was extended in the code to calculate degree centrality for groups. An example of an undirected network can be seen in Fig. 4a, and the calculated degree of centrality is shown in Fig. 4b.

### Distribution measure

Weighted degree centrality is a distribution measure implemented according to Candeloro et al. [26]. The given formulas were implemented in Python, by changing some aspects, and adding some details. Weighted degree centrality (WDC) can only be calculated for one node and is a measure of the uniformity of all outgoing edges from that node. To calculate WDC, one needs to know the number of outgoing edges (DC), which can be computed using <<Graph.out_edges(node)>>. Given the equations from the paper [26], the formula can be simplified in the following way:

(4) $$WDC=\;DC*\;\frac{AUC_{Fc}}{AUC_{max}}=DC*\;\frac{AUC_{Fc}}{\frac{DC}{2}}=2*\;AUC_{Fc}=1+2*\sum_{i=1}^{DC-1}Fc\left(i\right)$$

given that $\;AUC_{Fc}=\frac{1}{2}+\sum_{i=1}^{DC-1}Fc\left(i\right).$ The term Fc(i) in Eq. (4) is defined as a sum of edge weights. Given the definition in [26] it was calculated as

(5) $$Fc\left(i\right)=\;\sum_{J=1}^{i}\frac{w_{J}}{\sum_{k=1}^{DC}w_{k}}$$

with $w_{J}$ being the edge weight for an edge J.

#### Tips for application

When calculating WDCs for different participants but the same node described in Eqs. (4) and (5), the outgoing edges needed to be sorted by their weight size to compare uniform distributions between participants. This is not explicitly mentioned in the paper but becomes relevant if the first edge is not always the largest and there are different outgoing edges in different networks. So, before calculating WDC given the formula, the list of edge weights was sorted, $\left[w_{J},\;J\in \left[1,..,DC\right]\right].sort\left(\right)$. For an illustration, see Fig. 4c.

The uniformity measure is another distribution measure implemented using a chi-square test from the scipy.stats Python package [50]. This test for categorical data tests against the null hypothesis that the data is uniformly distributed (when using default arguments). To get a uniformity measure of gaze transitions, the chi-square test statistic can be calculated for all edge weights of a graph, multiplied by a negative one:

(6) U=(−1)*scipy.stats.chisquare(listofedgeweights).

As a result, the higher the U in Eq. (6), the more the gaze is uniformly distributed across the OOIs. A less uniformly distributed gaze network implies that participants often transitioned between a smaller subset of OOIs while ignoring other OOIs. Our analysis found that a smaller U was also correlated with longer fixation duration on frequently visited OOIs. However, the fact that some nodes are less frequently visited does not necessarily imply a smaller fixation duration for these OOIs. Participants could also focus on single nodes for a long time without transitioning much. For an illustration, see Fig. 4d.

### Interconnectedness measure

The cut size is an interconnectedness measure and is especially interesting when dividing the virtual space into different areas. The nodes of the graph can be separated into two unique groups. Cut size calculates the sum of all edge weights between these two groups. Cut size solely focuses on the connection between the two groups [41]. As a result, a larger cut size indicates more gaze transitions between the two groups, while a smaller cut size indicates less back and forth between the groups. An example can be seen in Fig. 4e.

#### Tips for application

One can either compute the cut size <<network.cut_size()>> or a normalized cut size with <<network.normalized_cut_size()>>, which is normalized to the sum of the total edge weights. The measures produce different results when comparing the gaze networks of different participants. When asked, “How often do participants transition between two sets of OOIs?” one should compute the cut size. In contrast, if the question is “Compared to all other gaze transitions in the environment, how much do participants transition between two sets of OOIs?” one should calculate the normalized cut size.

By computing cliques, other structural variables describing measures of interconnectedness could be calculated. A clique is the maximal subset of nodes, where all notes have edge weights larger than zero. This means at least one gaze transition must exist between all subset nodes. A node can be a part of different cliques, but different cliques must have at least one different (less/more/other) node.

The total number of cliques in one network reflects whether participants frequently transitioned with their gaze between objects. A higher number of cliques could be associated with less focused gaze behavior. The average clique size is another valuable variable since it captures the interconnectedness of information gained from cliques. Let us assume that one participant has a higher total number of cliques than another. One could not assume higher interconnectedness if one did not check for the average clique size because one large clique could collapse into two smaller cliques if, for example, one gaze transition was missing. An illustration of the structural variables calculated from cliques can be seen in Fig. 4f.

#### Tips for application

Cliques can only be computed for undirected graphs. Therefore, if the gaze-based attention network consists of directed gaze transitions, one must add respective incoming and outgoing notes and transform the graph into an undirected graph (UG). The networkx package has an implemented function <<network.find_cliques(UG)>>, which can be applied to UGs.

### Performance evaluation

To make this work more applicable to researchers, suggestions for efficient data processing were provided, and the performance of our data pipeline was evaluated. Data aggregation and network analysis, presented in Section 1.2, were evaluated based on the runtime metric. Specifically, to reach out to social scientists, who often use the programming language R instead of Python, the potential advantages and disadvantages of using R (Version 4.3.2) were evaluated. The tests and evaluations, as well as parts of the code programmed in R, can be found in our OSF repository (Ref. to OSF: 1-3_PerformanceEvaluation).

The eye-tracking datasets collected during the VR session differ in length, depending on the experiment duration. Additionally, the available hardware and the rendering complexity of the VR environment influence the framerate of the VR device and, therefore, determine the number of data points collected per second. For our experiment and data analysis, a Lenovo Legion 7 Laptop with an Intel Core i9-10980HK CPU @ 2.40 GHz, 3096 MHz, 32GB of RAM, and an NVIDIA GeForce RTX 2080 Super graphic card was used. One data point was obtained on average every 25 milliseconds (40 frames per second (FPS)). This resulted in an average of 36,000 data points for the 15-minute VR experiment for one participant (one minute ∼ 2400 data points). Note that remote eye trackers provide a higher temporal resolution (e.g., 1000 FPS), which cannot be compared to VR eye tracking and requires different processing and data handling.

The obtained dataset can be processed separately for each participant, so the total runtime would be the runtime of processing a single dataset times the number of participants. Similarly, the data pipeline saves newly aggregated, smaller data sets for each processing step, so the estimated runtime is the sum of all individual processing steps. The runtime for one representative dataset of one participant from the original sample was evaluated (dataset size: 38,005 rows). All runtimes of the following steps can be found in Table 2.

**Table 2:** Evaluated runtime of all processing steps of the data pipeline stated in seconds. Time is measured for one eye-tracking dataset (one participant). Potential runtime errors and how the data pipeline (method) avoids these are stated.

	Clean & create a smaller dataset	Create transition dataset	Create graph objects	Graph Features	
				Number of cliques	Weighted degree centrality
Python	0.58s	0.31s	0.01s	> 0.01s	> 0.01s
R	2.56s	0.51s	0.12s	0.01s	0.02s
Potential runtime errors	Loading large datasets can cause potential memory-related errors [51].	Filtering and processing data could encounter errors (missing values or incorrect conditions specified) [52].	Inconsistencies or unexpected data formats in the input data; dense or highly interconnected nodes [53].	Complex connectivity patterns or dense graphs may result in longer execution times or stack overflow errors [41].	
Solutions to runtime encounters	Saving and loading CSV files (and monitoring RAM usage).	Drop missing values and identify placeholders before processing; specify variable type.	Creating graphs from datasets with specified variables; dimensionality reduction by selecting or merging OOIs.	Avoid graph features that need extensive traversing through the graph. Keep the graph size low (see previous point).	

### Preprocessing

Working with large datasets, particularly in the context of eye-tracking data, can present challenges such as increased processing times, risk of stack overflow, and overall difficulties in data management. For researchers, especially those new to handling extensive eye-tracking datasets, performance optimization recommendations are offered in the subsequent steps (i.e., preprocessing, dimension reduction, and creating data duplications to offload tasks from primary memory storage (RAM) to secondary memory storage (CPU)) [51].

While some processing pipelines working with large datasets could encounter potential runtime errors [54], the design of our methods is specifically friendly to processing larger datasets. This is because the most memory-intensive task during data processing is parsing the raw data in the first step, while all subsequent steps profit from data reduction. Moreover, the network approach presents itself as a method to reduce data complexity and dimensionality [51], which was identified as a powerful tool in social sciences [55]. In the first step, it is advisable to narrow down the dataset to a smaller subset that contains only the essential variables required for your analysis (dimension reduction). Typically, datasets from VR experiments may include up to 90 different variables. However, for the methodology discussed in this paper, only two variables are essential (or five if pupil diameters are included for data cleaning). As described in Section 1.2 (I), the two essential variables are the time and the gaze target variable that contains the names of the OOIs collected via the ray-casting method. If pupil diameter is used for data cleaning, both left and right pupil diameter and, if available, eye-openness variables should be contained in the dataset for the first preprocessing step. Dropping unnecessary variables can lead to a reduction in dataset size by approximately 95%—for instance, reducing the dataset size from around 33 Megabytes (MB) to just 1.7 MB.

Additionally, these datasets often feature missing data or include OOIs irrelevant to the study. Eliminating these elements during the initial preprocessing steps results in more manageable dataset sizes and enhances processing efficiency in subsequent stages (preprocessing). In our experience, the time required for cleaning and saving a dataset for a single participant was 0.58 s in Python and 2.56 s in R. Additionally, processing each dataset separately and freeing memory after saving the data creates data duplications that offload task complexity from primary memory storage (RAM) to secondary memory storage (CPU) and distributes resources more evenly across hardware memory [56]. By adopting these strategies, researchers can significantly mitigate the computational challenges associated with large datasets and avoid potential runtime errors (Ref. to Table 2).

#### Aggregating raw gaze-target information into gaze transition datasets

For the preprocessed, smaller datasets, the initial step involves generating the transition datasets through a process that iterates over all rows in the dataset via a single ‘for loop.’ Consequently, the runtime of this operation exhibits a linear relationship with the size of the dataset (denoted as N), leading to a computational complexity of O(N) according to Big O notation. In terms of performance, this process took 0.31 s in Python and 0.51 s in R.

#### Creating gaze-based attention networks from gaze transition datasets

In the Python pipeline, an additional step was introduced to generate adjacency matrices compatible with the networkx package. This allowed the pipeline to construct graph objects directly from the data. Similarly, in R, the adjacency matrices were computed first, and these matrices were then used to create graph objects with the igraph library. The execution time for this process was 0.01 s in Python and 0.12 s in R. It's important to note that the dataset sizes progressively reduced through aggregation steps, resulting in a final graph that only includes OOIs from the environment as nodes, with the number of transitions between them as edges. In the performance evaluation, we deliberately chose not to filter out any OOIs, leading to a graph with 91 nodes, reflecting the 91 distinct gaze targets identified in the VR environment. This contrasts with the 26 nodes used in our original analysis. The number of OOIs, inherently constrained by the VR environment, thus determines the maximum size of our graphs.

#### Computing structural variables to describe gaze-based attention networks

Given the initial low runtime for graph creation, the runtime was only measured for two structurally complex variables: the calculation of clique numbers and weighted degree centrality. Consequently, R code was added to the repository for these calculations. The computation time for the clique numbers was less than 0.01 s in Python and 0.01 s in R. For weighted degree centrality, Python completed the task in less than 0.01 s, whereas R took 0.02 s. The performance of other structural variables, such as degree centrality, was not assessed because functions for these calculations are readily available in libraries like networkx (e.g., graph.degree()) and igraph (e.g., strength(graph)).

The analysis revealed no significant performance issues in either Python or R, with R consistently showing slightly longer runtimes for all tasks [57]. Despite the requirement for high-performance hardware to run VR experiments using the HTC VIVE, the data analysis procedures only necessitate the computational capabilities of standard hardware. This aspect underscores the efficiency of our methodology. While calculating eye movement features requires several iterations over the entire data set, this method allows the data to be condensed swiftly and efficiently into more manageable formats. The most performance-intensive aspect of our analysis is the initial data cleaning and reduction process.

### Considerations for implementation and application

For the method presented, some aspects should be considered for implementation. One important aspect is that the accuracy and precision of the integrated eye tracker affect the gaze-ray casting technique [10]. While the ray-casting technique collects data every time stamp, the measured information could be of varying quality. For example, if the OOIs in the environment are too small, the global gaze vector might miss the focused object. This can later be seen in the data when, for successive time stamps, different objects are tracked alternately. To avoid additional processing steps for data cleaning, the virtual environment should consist of larger OOIs. If this is not the case, one must consider merging smaller OOIs into bigger ones. The necessary size of an OOI can be determined by performing a test run before the experiment, where a test person should be asked to look at many smaller objects in the environment.

Another important aspect concerns the number of OOIs that are used in a network. The comparison of networks between participants by analyzing structural variables is not affected by the number of OOIs added to the network. Using a large number of different OOIs for the analysis might only influence the clarity of visualization. In contrast, when merging OOIs, the resulting networks might be under-complex. Imagine a network with only two large OOIs that is created by merging many smaller OOIs. Analyzing cliques would not be possible here because there would only be one clique, the trivial one. Moreover, two limitations must be formulated when using the method. First, it is important to note that our method only focuses on overt visual attention and does not cover peripheral perception (covert attention) or recognition aspects. A second limitation is that our method was only evaluated using a relatively static environment, where the OOIs did not move too much. The analysis of gaze-based attention networks in VR with moving objects might be more tentative. We recommend that future applications using our method investigate more dynamic virtual environments with moving objects. While analyzing moving scenes usually requires frame-by-frame object detection, our approach already incorporates this by using the physical objects as OOIs. Since the OOI position can be extracted from the gaze-ray casting pipeline, this information can be used to aggregate node feature information and be further processed.

Furthermore, the structural variables described in this article are only a selection of the measures that can be computed from graphs. Other measures could be considered to analyze gaze transitions. While degree centrality translates directly into a measure of attention distribution towards (groups of) OOIs, other centrality markers are closeness centrality, between centrality, or eigenvector centrality [58]. Depending on their calculation formula, these measures require different interpretations when applied to gaze-based attention networks. Most calculated variables can be used to perform statistical analyses. However, one must be aware that the values of the structural variables could be non-normally distributed.

Collecting node feature information can also extend our statistical analysis of structural variables using graphical neural networks (GNN) models. From our provided pipeline, the aggregated networks can be directly transformed into graph representations for GNNs with the pytorch.geometric [59] library. Some structural variables (like degree centrality or clique information) can even be incorporated as node features in the GNN. The presented network analysis can also be performed similarly with the R programming language [60]. Since R also has a data frame object type, the data can be transformed into graphs using, for example, the igraph package [61], which allows for the calculation of structural variables. Especially for statistical analysis and visualization, R might be a suitable choice. In contrast, state-of-the-art machine learning implementations using networks and graphs are provided in Python.

Our large empirical evaluation with *N* = 274 students showed that the method can be successfully applied and has proven that structural variables show great potential for analyzing students’ learning and social behavior in a VR environment. Future work should consider exploring the changes over time to examine the dynamics of gaze behavior within the given VR experience. The provided data structure also allows for modeling temporal graphs [62], [63], [64], which could be adapted in future research. Moreover, the full potential of this analysis might be revealed when applied to different VR settings and tasks, like visual exploration, navigation, or joint attention.

## Ethics statements

In this study, all procedures involving human participants were in accordance with the ethical standards of institutional and/or national research committees. Ethical approval for this study was obtained from the university's Ethics Committee prior to the beginning of the research. The Ethics Committee reviewed and approved all aspects of the study to ensure that they adhered to ethical principles and standards.

The study was conducted in accordance with the ethical guidelines set forth by the Declaration of Helsinki, as well as other relevant regulations and laws governing research involving human subjects. Participants were informed about the purpose and procedures of the study and provided written consent prior to their participation.

All data collected during the study were treated with strict confidentiality and anonymity to ensure that the privacy and well-being of participants were protected. Any identifiable information was removed or pseudonymized before the analysis.

## CRediT authorship contribution statement

**Philipp Stark:** Methodology, Software, Formal analysis, Writing – original draft, Writing – review & editing, Visualization. **Lisa Hasenbein:** Validation, Resources, Writing – review & editing. **Enkelejda Kasneci:** Conceptualization, Writing – review & editing, Supervision. **Richard Göllner:** Conceptualization, Resources, Writing – review & editing, Supervision, Funding acquisition.

## Declaration of competing interest

The authors declare that they have no known competing financial interests or personal relationships that could have appeared to influence the work reported in this paper.

## Data Availability

Data is shared on https://osf.io/pek4q/?view_only=ef151fd06ac8413a827020d4264b3c8d as part of the co-submission.

## Appendix Supplementary materials

Image, application 1
