# Supplementary material for: Gaze-based attention network analysis in a virtual reality classroom
Source: MethodsX. 2024 Mar 15;12:102662. doi: 10.1016/j.mex.2024.102662 (PMC10993185; doi:10.1016/j.mex.2024.102662)
Supplement: Supplementary file 1 [file mmc1.docx]

***Supplementary Material***

| 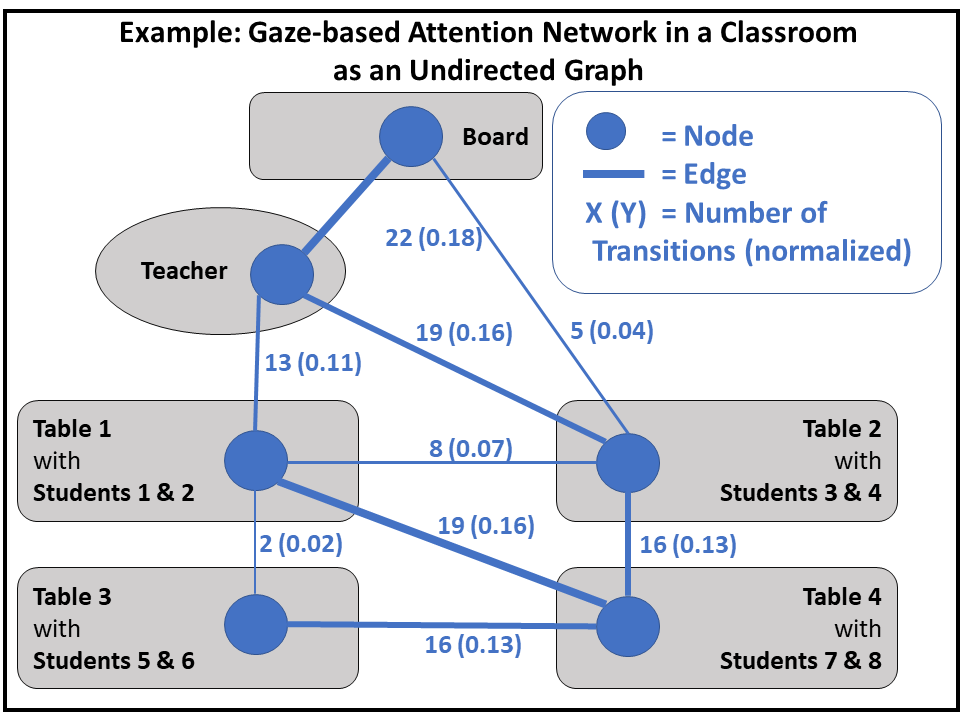  Figure 6a: Example network with all (normalized) numbers of transitions displayed as edge weights. Same image as in Figure 5a. |
| --- |
| 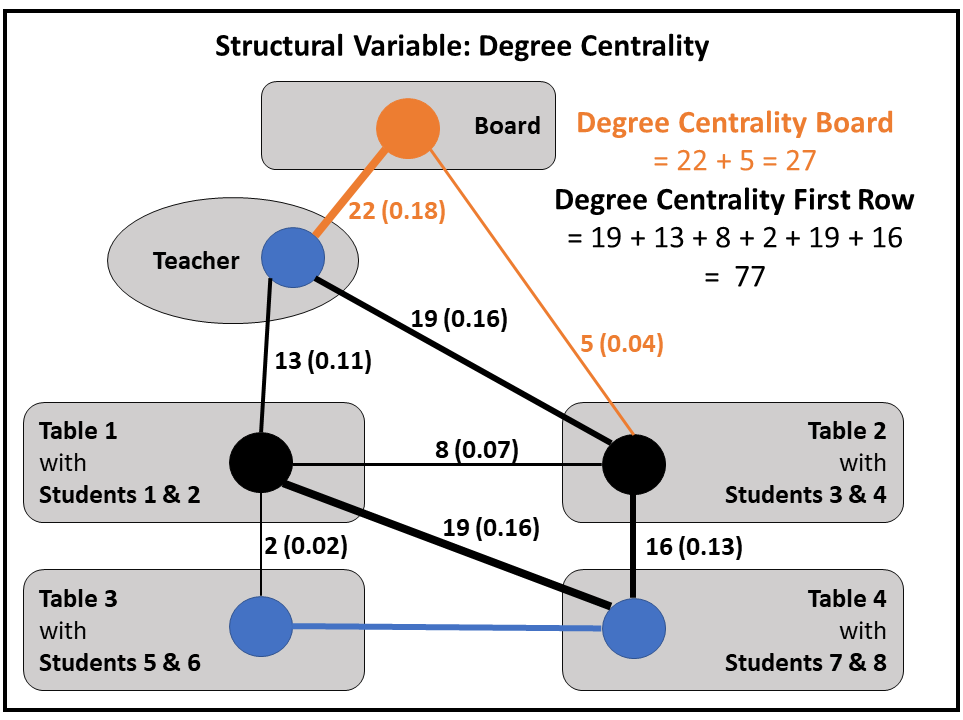  Figure 6b: Calculation of degree centrality for either a single node (board) or for a subset of nodes (first row of students). Same image as in Figure 5b. |

| 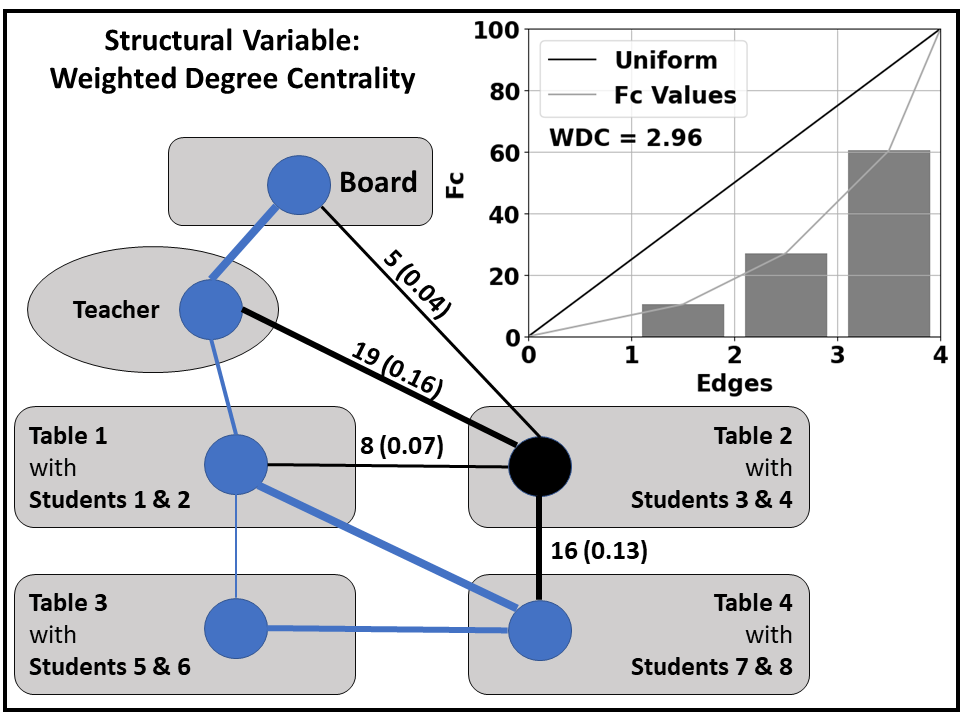  Figure 7a: Calculation of the weighted degree centrality for one node (table 2). The top right shows Fc calculated. Same image as in Figure 5c. |
| --- |
| 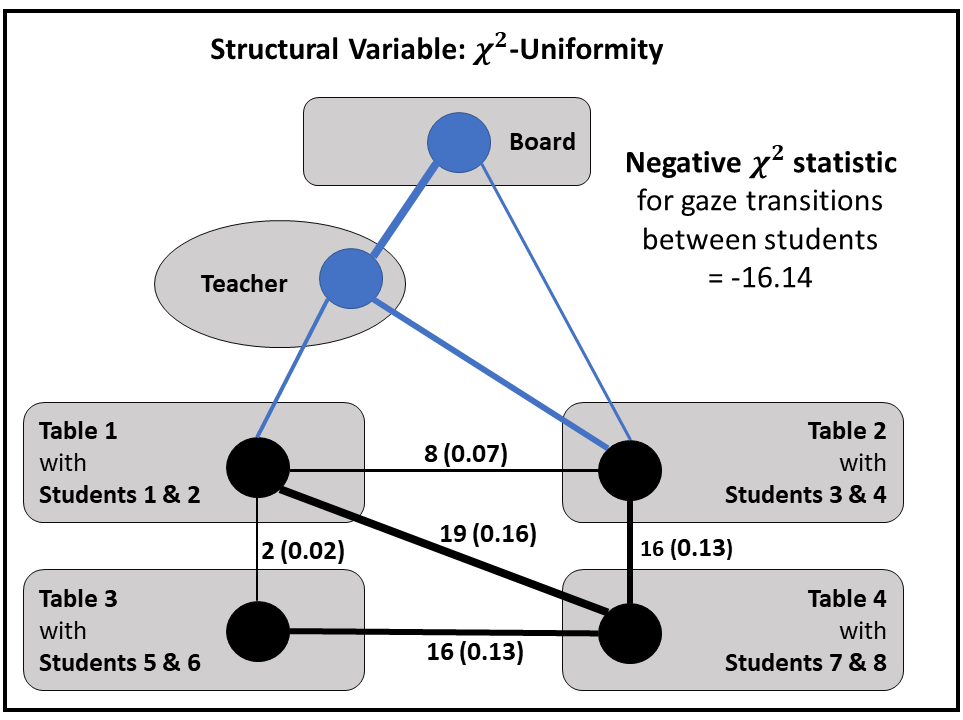  Figure 7b: Chi-square uniformity measure, calculated for a subset of all students. Not that this value is always negative due to the formula. Same image as in Figure 5d. |

| 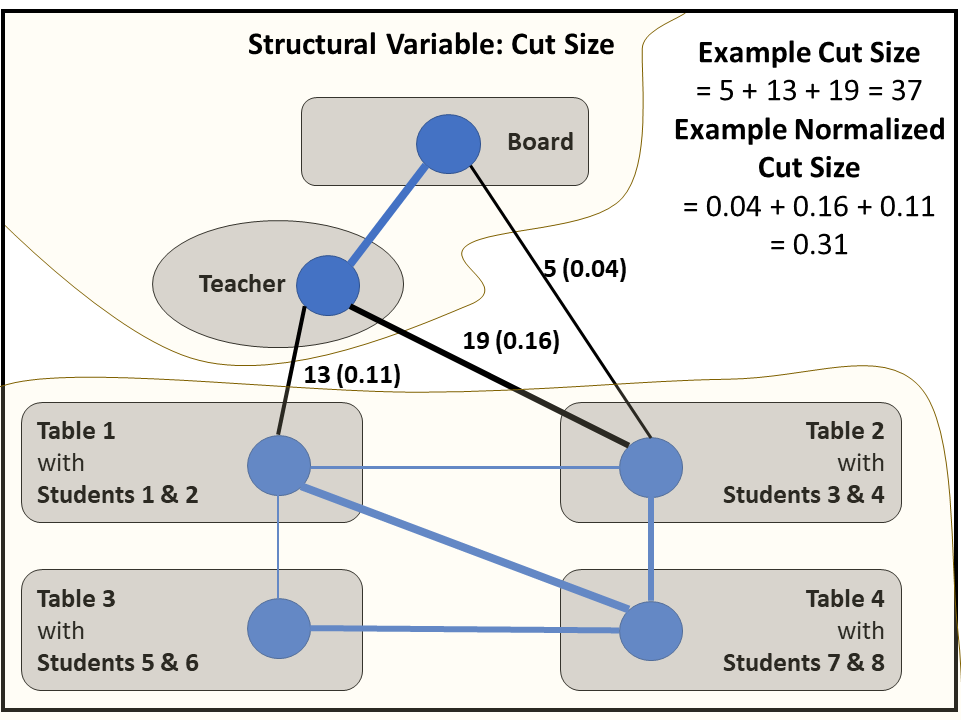  Figure 8a: An example of cut size when separating two subsets, namely all students and all non-students (teacher, board). Same image as in Figure 5e.  **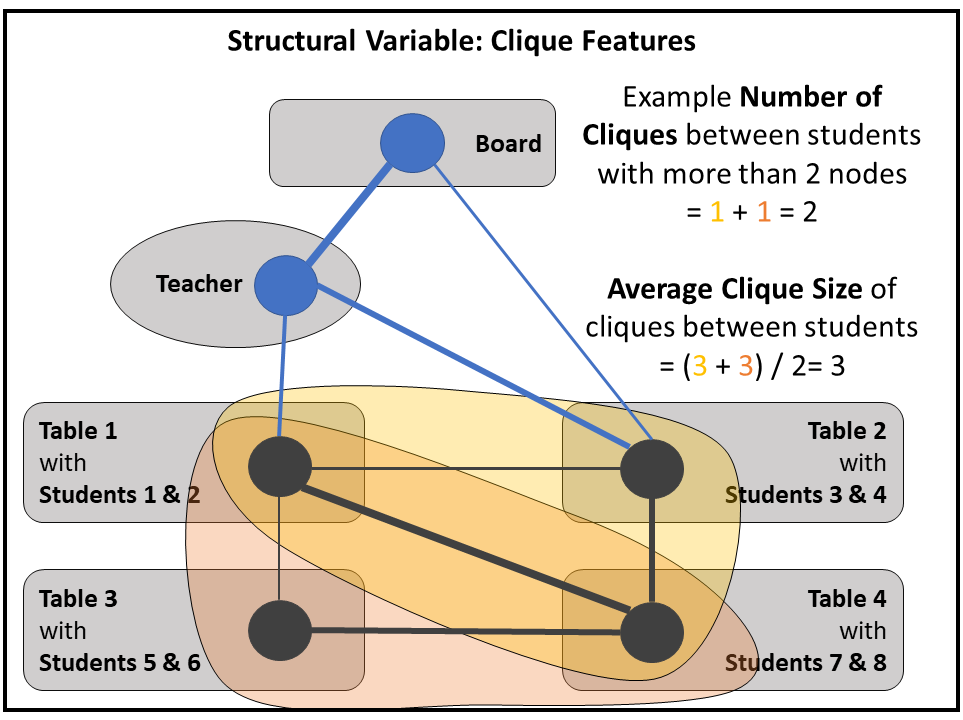** |
| --- |
| Figure 8b: Computation of all cliques of students larger than 2. The total number of cliques and average clique size can be calculated. Same image as in Figure 5f. |
